# Prestimulus vigilance predicts response speed in an easy visual discrimination task

**DOI:** 10.1186/1744-9081-7-31

**Published:** 2011-08-05

**Authors:** Juliane Minkwitz, Maja U Trenner, Christian Sander, Sebastian Olbrich, Abigail J Sheldrick, Peter Schönknecht, Ulrich Hegerl, Hubertus Himmerich

**Affiliations:** 1 Department of Psychiatry and Psychotherapy, University of Leipzig, Semmelweisstr. 10, 04103 Leipzig, Germany; 2 Department of Psychiatry and Psychotherapy, Medical Faculty, RWTH Aachen University, Pauwelsstr. 30, 52074 Aachen, Germany

## Abstract

**Background:**

Healthy adults show considerable within-subject variation of reaction time (RT) when performing cognitive tests. So far, the neurophysiological correlates of these inconsistencies have not yet been investigated sufficiently. In particular, studies rarely have focused on alterations of prestimulus EEG-vigilance as a factor which possibly influences the outcome of cognitive tests. We hypothesised that a low EEG-vigilance state immediately before a reaction task would entail a longer RT. Shorter RTs were expected for a high EEG-vigilance state.

**Methods:**

24 female students performed an easy visual discrimination task while an electroencephalogram (EEG) was recorded. The vigilance stages of 1-sec-EEG-segments before stimulus presentation were classified automatically using the computer-based Vigilance Algorithm Leipzig (VIGALL). The mean RTs of each EEG-vigilance stage were calculated for each subject. A paired t-test for the EEG-vigilance main stage analysis (A vs. B) and a variance analysis for repeated measures for the EEG-vigilance sub-stage analysis (A1, A2, A3, B1, B2/3) were calculated.

**Results:**

Individual mean RT was significantly shorter for events following the high EEG-vigilance stage A compared to the lower EEG-vigilance stage B. The main effect of the sub-stage analysis was marginal significant. A trend of gradually increasing RT was observable within the EEG-vigilance stage A.

**Conclusion:**

We conclude that an automatically classified low EEG-vigilance level is associated with an increased RT. Thus, intra-individual variances in cognitive test might be explainable in parts by the individual state of EEG-vigilance. Therefore, the accuracy of neuro-cognitive investigations might be improvable by simultaneously controlling for vigilance shifts using the EEG and VIGALL.

## Introduction

Typically, studies in cognitive neuroscience implement paradigms, e.g. cognitive performance tasks, in which participants respond to randomly presented sensory stimuli. By comparing averages of stimulus-locked responses, such as reaction time (RT) or error rate (ER), valuable information on cognitive processing can be gained. Beyond the variability between different subjects (inter-individual variability), responses of the same subject vary crucially (intra-individual variability) across experiments [1]. Previous studies report that inter-individual differences in RT are associated with gender, age [2] and neurological alterations [3-6].

High intra-individual variance of behavioural measures leads to biased evaluations of cognitive processing, both within healthy subjects as well as patient cohorts. Prolongation of an experimental paradigm is a frequently used and common method to reduce intra-individual variance for obtaining a more reliable measure of RT [7]. Nevertheless, the amount of experimental trials can be limited, e.g. due to reduced physical and mental condition of patients or older subjects. Thus, intra-individual variability should be minimised by controlling for potential covariates that directly influence RT and ER.

Therefore, we focused on the fluctuating state of wakefulness, as we hypothesized that the level of alertness crucially impacts individual performance during an experimental procedure. For examining our hypothesis, we adhere to the EEG-vigilance concept, which unfortunately overlaps with other concepts, e.g. alertness, attention and arousal [8]. We use the term *vigilance *to refer to different levels of brain function on the sleep-wake spectrum as they are empirically assessable by recording an electroencephalogram (EEG). Regarding specific EEG correlates of RT performance, Jokeit and Makeig [9] compared different EEG patterns of subjects with quick and slow mean RT. Qualitative differences in EEG patterns were reported between these two subject groups. Examining EEG patterns of healthy subjects, Delorme et al. [10] have revealed that larger low-theta complexes precede quicker motor responses both within and across subjects. Moreover, Makeig and Jung [11] demonstrated that performance variations on an auditory vigilance task show distinct EEG-correlates on different time-scales.

**Figure 1:**
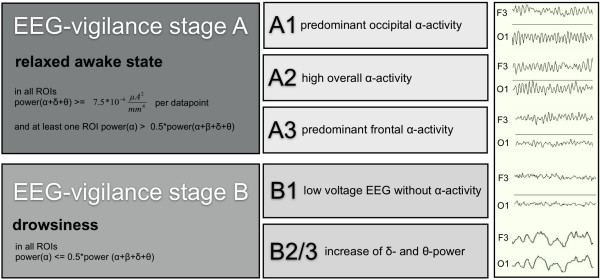
**EEG-based definition criteria of VIGALL for vigilance classification according to Bente (1964) & Roth (1961)**. Note: Vigilance stages were sub-classified (column 2) according to Bente (1964) and Roth (1961). Continuous EEG-based vigilance stages from full alertness to drowsiness are determined by VIGALL according to defined decision criteria (column 1). The first column presents that vigilance stage A is corresponding to the presence of high alpha power. Low alpha power features vigilance stage B. VIGALL classifies substages based on EEG-power source estimates using sLORETA: A1 (occipital ROI power (α) > = parietal and frontal ROI power(α)), A2 (occipital ROI power (α) < parietal and frontal ROI power(α) and temporal and parietal ROI power(α) > = frontal ROI 1.5* power (α)), A3 (occipital ROI power (α) < parietal and frontal ROI power(α) and temporal and parietal ROI power(α) < frontal ROI 1.5* power (α)), B1 (power(α+δ+θ) in one ROI = < 7.5*10^-6 ^μA^2^/mm^4 ^per data point), B2/3 (power(α+δ+θ) in one ROI > 7.5*10^-6 ^μA^2^/mm4 per data point. The right column depicts EEG curves of native two-seconds-segments.

The EEG is the only non-invasive method that directly measures neuronal activity with sufficient time resolution. On the basis of specific EEG-patterns, Loomis [12], Bente [13] and Roth [14] classified different activation states of the brain on a continuum reaching from the concentrated awake state to the state of deep sleep. In the following, these states, which influence the ability to process information, are termed EEG-vigilance stages. They have been carefully described and subdivided (A1, A2, A3, B1, B2/3) depending on the frequency and topographic distribution of the EEG-waves (see Figure 1).

Nevertheless, examination of EEG-vigilance based variability in RT tasks has not been used on a single-trial basis so far due to methodological difficulties. The monitoring of fluctuating vigilance by parameters of the peripheral nervous system, such as heart rate and electrodermal activity (EDA), proved to be unreliable due to the slow response rates of these indirect parameters. Thus, the assessment of vigilance via EEG appears to be an adequate approach to determine global functional levels of the brain. However, even expert raters showed poor performance in identifying vigilance lapses using EEG [15]. Therefore, Hegerl et al. [16] developed a computer-based algorithm (VIGALL, Vigilance Algorithm Leipzig) that classifies different vigilance stages of EEG segments according to Bente and Roth (A1, A2, A3, B1, B2/3), based on the frequency and topographical distribution of the neuroelectric activity. Olbrich et al. [17] validated and refined this algorithm. Hence, VIGALL is now based on EEG-power source estimates using LORETA (Low Resolution Brain Electromagnetic Tomography) and enables the classification of EEG-vigilance stages for 1-sec-segments. Figure 1 depicts decision criteria of the algorithm to calculate vigilance stages from the EEG data obtained.

The goal of the present study was to determine whether the VIGALL-classified prestimulus state of EEG-vigilance is associated with the length of RT and may therefore explain the intra-individual variance of this dimension. We postulated that a low prestimulus EEG-vigilance state (B-stages) leads to longer RTs and that a high vigilance state (A-stages) entails shorter RTs. Additionally, we intended to conduct an explorative analysis of the relationship between the EEG-vigilance substages (A1, A2, A3, B1, B2/3) and RTs.

## Methods

### Participants

To reduce the inter-subject variability to the greatest possible extent, a homogenous group of healthy female students, who had undergone an extensive screening for somatic and mental disorders, was included in this study. In total, 35 female students from 20 to 30 years of age (M = 23.71, SD = 2.78) participated in the investigation. These volunteers were recruited through advertisement and received remuneration. All participants reported no psychiatric, neurological or serious medical conditions. Physical health was screened in a semi-structured interview and mental health was examined according to the criteria of the diagnostic and statistical manual of mental disorders (DSM-IV) by applying a German version of the Structured Clinical Interview for DSM-IV disorders (SKID-I) [18]. In order to exclude subjects currently abusing alcohol and drugs, general alcohol and drug consumption was quantified by administering the alcohol use disorders identification test (AUDIT) [19] and the drug use disorders identification test (DUDIT) [20]. All subjects reported normal or corrected-to-normal visual acuity.

A total of 11 subjects had to be excluded post-hoc from the main stage analysis (EEG-vigilance stage A vs. B). Reasons and a description of the exclusion procedure are specified in the data preparation section. The final sample comprised 24 female students with an age-range from 20 to 29 years (M = 23.54, SD = 2.67) for the EEG-vigilance main stage comparison and 5 females from 21 to 29 years (M = 24.60, SD = 3.29) for the explorative comparison of RTs of the EEG-vigilance substages. A local ethics committee approval and written informed consent from each volunteer were obtained prior to the investigation according to the declaration of Helsinki.

### Measures and procedure

Subjects performed a 15-minute visual discrimination task. Simultaneously, an EEG was recorded in a dimmed and sound attenuated room. Participants sat in a comfortable chair in an upright position. To avoid circadian effects, all EEGs were performed in the middle of the afternoon.

#### Cognitive performance test (CPT)

The cognitive performance task used in this investigation covered 400 randomized trials. The visual stimuli consisted of bold white letters with a width of about 9 cm and a height of about 10 cm which appeared on a black background. The stimuli set contained the target "X" in 70% of the trials, and the distractor "O" in 30% of the cases. Each stimulus was presented for 300 ms on a computer screen in front of the sitting participants with an inter-stimulus-interval of 2000 ms. The subjects' distance to the monitor was approximately 120 cm. The subjects were instructed to press a button with the index finger of their dominant hand in case of target presentation. Due to the fact that the applied visual discrimination task is very easy with only two different stimuli, we expected that the rate of hits (correctly detected targets) would be high while the rate of errors, including false alarms and misses, would be low. For this reason, our analysis focussed on the variability in RT, not on precision (ER).

#### EEG procedure

##### EEG set-up and recording

31 electrodes (sintered silver/silver chloride) placed according to the 10-20 international system with impedances kept below 10 kOhm were applied to record the EEG. Data was recorded with a 1 kHz sampling rate and common average was used for reference. Additionally, an electrocardiogram (ECG) and electrooculogram (EOG) were recorded to control for cardial and ocular artefacts. For EOG-recording, one electrode was taped on the forehead and a reference electrode was fixed on the cheek below the eye. ECG electrodes were placed on the right and left wrist. The recordings were amplified by a 40-channel-QuickAmp unit and BrainVision 2.0 software (BrainProducts, Gilching, Germany), which was installed on a Microsoft Windows XP compatible computer system, was used.

##### Pre-processing of EEG data

EEG data was pre-processed with the Analyzer software package according to the following steps. First, EEG raw data was filtered at 70 Hz (low-pass), 0.5 Hz (high-pass) and 50 Hz (notch-filter, range 5 Hz). Study-relevant EEG-segments were cut out including two 1-sec-segements before and after the relevant segments prior to target presentation. This ensured that on- and offset effects of subsequent analysis steps were avoided. Then, the independent component analysis (ICA)-based approach [21,22] was used for both the removal of eye artefacts and the correction of EEG-channels with continuous muscle activity. After segmentation into consecutive one-second intervals, data sets were again screened for remaining muscle, movement, eye and sweating artefacts. Those artefacts were marked for exclusion from the EEG-vigilance stage analysis. Afterwards, complex demodulation of the EEG-frequency bands 2-4 Hz (delta), 4-8 Hz (theta), 8-12 Hz (alpha) and 12-25 Hz (beta) were computed for all EEG channels to obtain the frequency band envelope magnitude in μV^2 ^in order to approximate the power of the underlying signal [23].

Using the LORETA module of the Vision Analyzer software, the intracortical averaged squared current densities of frequency band power in four predefined regions of interests (ROIs) were calculated. The term averaged current densities refers to 1) the spatial averaging of the electrical intracortical source estimates of each voxel included within the four regions of interest in occipital, parietal, temporal and frontal cortices and 2) the temporal averaging of the current densities at all data points within a one-second segment (i.e. 100 data points for a sampling rate of 100 Hz). The occipital ROI involves the occipital lobe and the cuneus, because alpha activity during rest is most prominent in those areas [24]. The parietal ROI consists of the superior and inferior parietal lobe, where shifts of alpha power have been found during the transition phase from full wakefulness to sleep [25,26]. The temporal ROI comprises the inferior temporal lobe owing to most prominent EEG-alpha power in the inferior lobe during light sleep stages [27]. The frontal ROI consists of the anterior cingulate gyrus (ACC) and the medial frontal gyrus as the most prominent EEG alpha power and EEG theta power during drowsiness is located within these areas [28,29].

##### Classification of EEG vigilance stages using VIGALL

According to EEG-source estimates in the ROIs, EEG-vigilance stages were classified by the VIGALL algorithm (Figure 1). The used intracortical current source density thresholds for stage B1 correspond to the topographical cut-off criterion of 200 μV^2 ^for Fast-Fourier transformed EEG-data at channels F3-TP9, F4-TP10, O1-TP9 and O2-TP10 as it has been used in the study by Olbrich et al. [17]. Reanalysing the EEG data from this study, it was found that the current source densities within the ROIs did not exceed the reported threshold for stages classified as stage B1. Also the reported proportions for e.g. alpha anteriorisation (stage A1-A3) correspond to the topographical distribution of EEG-power that has been used in the former version of the algorithm. However, the EEG-vigilance substages were subsumed under main stage A (A1, A2 and A3) and B (B1 and B2/3) for the analysis of RT differences between high and low vigilance states. Lower vigilance stages than B2/3, characterised by K-complexes and sleep spindles, did not occur within data sets. For statistical analyses, only the vigilance stages that occurred 1 sec prior to target presentation were evaluated.

### Data preparation

Four data sets had to be excluded due to lacking quality of the recorded EEGs: In two cases, the raw data contained more than twenty percent of segments with artefacts; another two recordings had no impedance information and were excluded for this reason.

**Figure 2:**
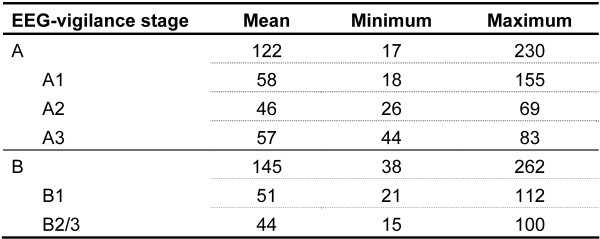
**The number of single trials for each vigilance (sub-) stage**.

Since an unbalanced distribution of vigilance stages (e.g. the exclusive presence of one vigilance stage) would make it unfeasible to test the study hypotheses, a minimum of vigilance variability within the same individual is necessary. For this reason, a minimum of 5% of each vigilance (sub-) stage was set as a further inclusion criterion for the comparability of RT differences. Therefore, seven subjects had to be excluded for the comparison of RTs of stage A with RTs of stage B (EEG-vigilance main stage analysis). Hence, 24 subjects were included in further main stage analysis. Only five subjects displayed at least 5% of each EEG-vigilance substage (A1, A2, A3, B1, B2/3) and were included in the additional explorative analysis of EEG-vigilance substages. Figure 2 features descriptive information concerning the number of trials of each vigilance (sub-) stage. According to the assumption of low ERs owing to the simplicity of the applied CPT version, no participant had to be excluded because of too many faults (M = 1.69, SD = 1.38, range 0.36-5.40).

VIGALL classifies 1-sec-segments of the EEG data prior to target presentation separately for each trial and subject. RTs of trials with the same vigilance classification were averaged, thus mean RTs for the different EEG-vigilance (sub-) stages are available for each subjects.

Extremely fast or slow responses were treated as missing values as they potentially reflect errors such as key malfunctions or accidental keystrokes. The computation of the mean RTs comprised all trials with response times between 200 ms and 1000 ms. Missing values also resulted from non-classifiable EEG segments owing to artefacts. In total, between 234 and 280 (MW = 266.46, SD = 12.35) responses were used to compute the mean RTs of the subjects.

### Statistics

All data were processed using the PASW Statistics 18.0 Package for Windows. The hypothesis that vigilance influences the speed of reaction was examined by applying a paired t-test for the EEG-vigilance main stage analysis (A vs. B) and a variance analysis for repeated measures for the EEG-vigilance substage analysis (A1, A2, A3, B1, B2/3). Hypotheses were tested two-tailed. A probability p value of less than 0.05 was considered significant, whereas marginal trends were determined up to a significance level of 0.10. Normal distribution was tested by the Kolmogorov-Smirnov test. All variables were normally distributed.

## Results

### Vigilance

The distribution of the different states of wakefulness was determined for each participant. Overall, B-stages (M = 54.33%, SD = 26.11) were registered slightly more frequent than A-stages (M = 45.67%, SD = 26.11), however, this was not statistically significant (t(23) = -0.812, p = 0.425).

### Vigilance and RT

**Figure 3:**
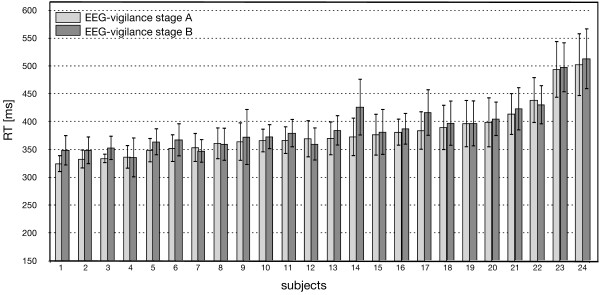
**Individual RT (n = 24) referring to the main EEG-vigilance stages A and B**. Note: The error bars represent the individual standard deviations.

The mean RT was calculated individually for all participants for each of the main vigilance stages. A paired t-test was used to assess the difference between response times of the two different conditions (vigilance stage A vs. B). The difference between the individual RT in the vigilance stages A vs. B was statistically significant (t(23) = -2.805, p < 0.05). Individual mean RTs were significantly shorter for events following high EEG-vigilance stage A (M = 380.60 ms, SD = 44.91 ms) compared to the lower EEG-vigilance stage B (M = 388.37 ms, SD = 44.15 ms). For the individual RTs of every volunteer see Figure 3.

**Figure 4:**
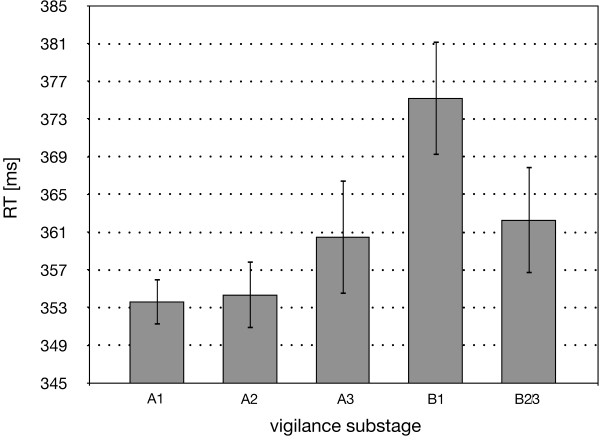
**Mean RT referring to the EEG-vigilance substages (n = 5)**. Note: The error bars represent the standard error of the mean.

For the explorative analysis of the relationship of EEG-vigilance substages and RT, an ANOVA for repeated measurements (5 levels: A1, A2, A3, B1, B2/3) was processed. Results (F(4;16) = 2.643) of the ANOVA provided marginal significance (p = 0.072) for the main effect EEG-vigilance stage. The substages of the main EEG-vigilance stage B entailed longer RTs than the substages of the EEG-vigilance stage A. Furthermore, a trend of gradually increasing RT was observable within the EEG-vigilance stage A (see Figure 4).

## Discussion

In accordance with our hypothesis, we found that in a continuous performance task, RT depends on the prestimulus EEG-vigilance stage. Faster individual reaction was observed during a higher EEG-vigilance level (stage A), whereas a declining response speed was detected for lower states of EEG-vigilance (stage B). Furthermore, marginal differences in RTs between the EEG-vigilance substages were found despite the small sample size of this subgroup. However, a continuous increase in RT with decreasing vigilance levels was only found for the substages A1, A2 and A3. Decline of vigilance from substage B1 to B2/3 did not yield the expected increase in RT.

A reason for the latter result might be that stage B2/3 is defined as an EEG-vigilance stage with low alpha power but high delta and theta power. Especially, increased phasic theta power has been associated with cognitive performance during cognitive tasks [30]. In contrast to this, frontal theta power also increased during rest without mental occupation as a sign of a further decline of vigilance [31]. VIGALL originally was intended for classification of EEG-vigilance stages during rest and hence it is possible that stages with high theta power during the cognitive performance test within this study were misinterpreted as low subvigil stages B2/3 although they might reflect a higher vigilance stage. As a consequence, RT of substages B2/3 show decreased RTs in comparison to stage B1. Another explanation for this inconsistency of RT alteration with changing EEG-vigilance substages might be the small sample size or a lacking sensitivity of the computer-based vigilance algorithm under the condition of open-eyed-EEG recordings.

Previous studies assessing factors influencing RT variability either primarily focused on patient samples [5,6] or identified variables that describe differences between subjects, for instance gender and age [2]. Thus, the influence of transient within-subject factors on cognitive performance tests, such as fluctuations in motivation or wakefulness, has not been considered adequately in previous studies. Nevertheless, an early examination by Lansing et al. [32] described the influence of alertness, determined by patterns of alpha rhythm in EEG, on RT. The authors showed that subjects displayed faster RTs in the alerted than in the non-alerted condition. These results are consistent with our findings of shorter RTs in case of high-vigilant states. However, the experiment deviated from our methods as alertness was induced by alarm signals. We determined the vigilance state without exerting an influence on vigilance shifts. Moreover, in our study the classification of vigilance states was computed automatically by the EEG-based algorithm VIGALL, whereas certain EEG-patterns in the previous study were detected by individual raters. Hence, our method to classify vigilance is certainly more economic and reliable and might therefore be broadly applicable in future measurements.

Also, in a study on the coherence of fluctuations in performance and EEG spectrum, Makeig and Inlow [33] reported highly positive correlations between EEG power below 6-7 Hz, error rate and highly negative correlations near 10 Hz. The finding of increased ERs during the appearance of delta and theta waves in the EEG corroborate our results that performance becomes poorer during lower states of vigilance. The observations by Makeig and Inlow are in agreement with Jung et al. [34], who reported a correlation between increased ER and EEG power below 5 Hz.

Hultsch and colleagues [5] suggested that intra-individual variability might be a marker for impaired neurological functioning, as patients with mild dementia showed twice as much intra-individual variability in performance as neurologically intact participants. Therefore, monitoring of factors which cause within-subject variations during a performance task is essential to evaluate the observed individual variability. As demonstrated in the present study, the covariate vigilance explains the variance to a certain extent. Vigilance-specific alterations of electric brain activity during performance tasks should thus be taken into account. By disregarding individual vigilance-driven electrical brain signal fluctuations, relations between intra-individual variability of RTs and neurological disease might be either over- or underestimated.

Hence, the present study is methodologically important by emphasising the necessity of considering vigilance in studies whilst planning, performing and interpreting cognitive tasks. We determined vigilance using EEG, a well-established and non-invasive method in medicine. By this method, vigilance monitoring and classification is broadly applicable in future studies to control for intra-individual variability.

Furthermore, the observation that vigilance affects behavioural measures opens up perspectives to further improve the validity of neuro-imaging methods. For instance, functional imaging studies might profit from eliminating unexplained intra-individual variance by taking different states of vigilance into account. Therefore, simultaneous usage of functional imaging methods and EEG is beneficial. Technical requirements have already been met and PET- and fMRI-compatible EEG instruments are now available. Thus, the covariate vigilance can be controlled for by monitoring for vigilance shifts using the VIGALL algorithm during neuro-imaging procedures. When analysing and interpreting neuro-imaging data sets, EEG-vigilance stages could either be considered in the general evaluation, or only those time segments could be taken into account that show certain vigilance states. Further studies are needed to assess the general impact of vigilance states on neuro-imaging methods.

Despite these advantages of the presented study, some limitations also have to be mentioned. One shortcoming of our examination is the fact that, for reasons of minimising the inter-individual variability, a rather homogeneous group of healthy female students was included in the study. It remains uncertain whether the findings can be transferred to other cohorts. Investigations with different samples are required to validate the observed influence of vigilance states on RTs. In addition, the sample size of 24 healthy participants was small. This statistical drawback becomes more noticeable for the analysis of the vigilance substages, as the sample size for the explanatory ANOVA analysis is very small with only 5 subjects included. The vigilance effect should be validated by future studies with larger numbers of patients and healthy controls. Furthermore, as only an easy visual discrimination task was carried out, the obtained findings can not be generalized for all RT paradigms. According to Stuss et al. [35], RT variability might increase in more complex tasks. Therefore, several RT tasks involving different qualities of sensory perception and various levels of difficulty should be implemented. Another drawback of the study is that the used version of VIGALL could not be adjusted individually, as it used equal EEG criteria for all participants. Currently, the VIGALL team is refining the operating mode of the algorithm taking into account the individual alpha peak instead of fixed frequency windows for VIGALL processing. Consequently, individual boundaries of frequency bands could be justified, making the vigilance classification of VIGALL more exact.

Of course, vigilance is only one possible factor which might influence RT in real life. For example, age [36], alcohol [37], drugs [38], certain psychiatric [39] and somatic diseases [40] as well as distraction [41] have been shown to influence RT. Our results suggest that hormones which influence the sleep-wake regulation and therefore vigilance such as glucocorticoids, melatonin and leptin as well as other hormones which influence these endocrine systems such as estrogens, androgens and thyroid hormones might also play a role as influencing factor on reaction time. Therefore, we are going to investigate the influence of these hormones on RT and differences regarding influencing factors on RT in females and males in future studies. Due to fact that this is a pilot study using a small group of homogenous healthy female subjects, we have to discuss the limitation that we are not able to give any data regarding these mentioned additional possible influencing factors such as age, gender, alcohol, drugs, psychiatric and somatic diseases, distraction and hormones. Another important issue to consider is specific methodological approach of this study. The applied CPT was very easy and monotonous, since we intended to induce a vigilance decline. The inter-stimulus-interval was 2000 ms. Furthermore, the smallest possible VIGALL analysis unit is one second. We decided to analyze the whole second before target presentation and not while or after target presentation, because a) the stimulus presentation induces an arousal and modifies the EEG and b) the subjects' reaction leads to artefacts in the EEG and is expected 300-500 ms after target presentation. Therefore, our results regarding vigilance are-with good reason-limited to the one second before target presentation.

In summary, the results of the present study demonstrate that RT is associated with prestimulus vigilance as it can be measured using EEG. By including this EEG-measured vigilance in the analysis, the conclusiveness of scientific data on cognitive performance or reaction tasks might be improved. This may be relevant for neuropsychological as well as for functional neuroimaging studies.

## Competing interests

All authors declare not to have any conflict of interest including any financial, personal or other relationships with other people or organizations that could inappropriately influence, or be perceived to influence, their work.

## Authors' contributions

The presented work was carried out in collaboration between all authors. All authors were involved in drafting and revising critically the manuscript and approved the final version for publication. PS and UH defined the research theme and planned the conception of the study. MT and CS designed the experiment methods and acquired data. JM and HH made substantial contributions to the conception and design, analyzed the data, interpreted the results and wrote the paper. SO and AS directed the EEG recording methods and discussed analyses, interpretation and presentation.
